# Sleep, alcohol, and caffeine in financial traders

**DOI:** 10.1371/journal.pone.0291675

**Published:** 2023-11-08

**Authors:** Frank Song, Matthew P. Walker

**Affiliations:** 1 Center for the Study of Health and Risk Behaviors, Department of Psychiatry, University of Washington, Seattle, Washington, United States of America; 2 Center for Human Sleep Science, Department of Psychology, University of California, Berkeley, California, United States of America; Charité - Universitätsmedizin Berlin, GERMANY

## Abstract

Alcohol and caffeine are two of the most commonly used substances for altering human consciousness. While their adverse effects on sleep have been separately examined in the laboratory and epidemiological levels, how they impact real-world night-to-night sleep, in isolation or together, remains unclear. This is especially true in occupations wherein the use of alcohol and caffeine is high (e.g., financial services sector). Using a six-week micro-longitudinal study, here we examined the real-world impact of alcohol, caffeine, and their combined consumption in a cohort of financial traders. We demonstrate that alcohol consumption significantly degrades the subjective *quality* of sleep (*p* < 0.001). Caffeine consumption led to a different phenotype of sleep impairment, resulting in a detrimental reduction in sleep *quantity (p =* 0.019*)*, rather than a marked alteration in sleep quality. Contrary to our hypothesis, when consumed in combination, evening alcohol consumption interacted with ongoing caffeine consumption such that alcohol partially mitigated the impairments in sleep quantity associated with caffeine (*p =* 0.032). This finding suggests the sedating effects of alcohol and the psychoactive stimulant effects of caffeine obscure each other’s impact on sleep quantity and sleep quality, respectively–potentially explaining their interdependent use in this cohort (i.e., “self-medication” of evening sedation with alcohol to combat the prior daytime ingestion of caffeine and vice versa). More generally, these results contribute to a unique understanding of the singular and combinatory impacts of two of the most commonly used substances for augmenting human consciousness under free-living, real-world conditions, the performance-impairing (and thus economic-cost) consequences of which may be important to the business sector and the society.

## Introduction

Alcohol and caffeine are two of the most widely consumed psychoactive substances in the world. Moreover, they are the two most commonly used substances for manipulating human consciousness, most notably sleep and wakefulness. In 2016, alcohol consumption exceeded 6.4 liters per person aged 16 or older globally, or the equivalent of drinking a liter of wine each week [1]. The global average caffeine consumption was 530mg per person per week, equating to 5.5 cups of coffee [2].

While the prevalence of alcohol consumption differs by country, in the 30 countries with the highest prevalence (representing over 1 billion people), 74.1% of people above the age of fifteen report drinking alcohol in varying degrees [1]. Regarding caffeine consumption, approximately 90% of all adults worldwide consume caffeine daily, primarily by drinking caffeinated beverages such as tea or coffee [3–5]. Numerous experimental reports to date have described the adverse effects of both alcohol and of caffeine on human sleep. Alcohol is robustly associated with impairments in sleep quality, including increases in nocturnal awakenings and reductions in sleep efficiency and the amount of rapid eye movement (REM) sleep [6–9]. Moreover, these alcohol-related impairments in sleep are associated with consequential impairments in daytime functioning, including working memory capacity [10, 11], decision making [12], and ability to sustain attention [13]. Caffeine is especially associated with impairments in sleep quantity, both by way of delaying the ability to fall asleep (i.e., longer sleep latency) and reducing the ability to generate sleep, leading to a reduction in total sleep duration [14–18]. Moreover, caffeine consumption is associated with a reduction in the quantity of deep non-REM (NREM) slow-wave activity [17, 19].

While these studies describe the effects of either alcohol or caffeine in isolation, the combined effects of consuming both substances is considerably less well understood. In controlled laboratory studies, drinks that contain both alcohol and caffeine have been linked to a four-fold increase in the likelihood of reporting an inability to sleep [20]. Drinking alcoholic beverages in combination with consuming caffeine has further been associated with prolonging the time it takes to fall asleep, relative to drinking alcoholic beverages alone [21]. In addition, alcoholic beverages containing caffeine have been associated with increases in night-time awakenings, compared with drinking only alcoholic beverages [22]. Interestingly, however, one study has reported *improved* (subjective, at least) sleep quality following the combined consumption of alcohol and caffeine, relative to consuming only alcohol [8]. However, post-hoc tests indicated that this increase in subjective quality was the result of improved alertness upon awakening, and not superior quality of sleep the night prior [8].

While these experimental studies have productively enhanced our knowledge regarding the effects of alcohol and caffeine, and to some degree, their combination, the laboratory setting poses certain limitations in generalizing results to real-world settings, especially in professional occupations where heavy use of both caffeine and alcohol are common, including high-stakes finance [23–25]. Furthermore, no study to date has assessed the effects of consuming alcohol and caffeine within the same individuals in a longitudinal manner, using multiple time points. The latter is especially critical considering the common real-world practice of repeated joint caffeine and alcohol consumption each day, across days. This is important considering that the intra-individual variability with which alcohol, caffeine, and their combined use vary considerably, which would be expected to change the interaction effects on consequential sleep on those following nights.

Here, we sought to address these unresolved questions. We specifically tested the hypothesis that the disruptive effects of caffeine and alcohol on sleep are both observable under real-world professional operating conditions, and moreover, that these relationships manifest in a temporally interacting and combinatorial day-to-day, night-to-night manner, within the same individuals over time. We tested this hypothesis in a group of professionals where degraded sleep quality is known to have a deleterious impact on performance [26], and the combined consumption of alcohol and caffeine is common: that of financial traders [23–25].

The experimental hypothesis was tested using validated digital daily surveying tools that quantified alcohol and caffeine consumption habits and sleep-related measures over a six-week (42 days) period as individuals went about their lives during their work weeks and weekends. The latter comparison is relevant considering the known weekday vs. weekend differences in both sleep and alcohol consumption [27–30]. Their work weeks consisted of daytime hours from Mondays through Fridays, approximately between the hours of 8:30 a.m. and 5:30 p.m. During pre-study interviews, they consistently reported regular daytime use of caffeine and night-time use of alcohol in their daily lives.

Building on this overarching hypothesis, we sought to test three specific predictions: 1) the consumption of caffeine decreases the ability to generate sleep, resulting in a reduction in sleep amount (quantity) and degradation of subjective quality of sleep, 2) alcohol consumption adversely impacts quality of sleep that following night and increases the number of night-time awakenings, reflecting sleep fragmentation, and 3) concerning the combinatorial effect, the subsequent night-time consumption of the sedating compound of alcohol would diminish the otherwise alerting effect of prior daytime caffeine ingestion and its impairment of sleep quality, and that these effects would be additive or multiplicative, relative to either caffeine or alcohol consumption alone on any given day. In addition to testing these primary hypothesis predictions, given evidence to suggest a bidirectional association between substance use and sleep problems [31, 32], we also sought to assess the inverse hypothesis effects: that is, whether sleep quality and quantity affect the next day’s caffeine and alcohol consumption decisions on a day-to-day/night-to-night basis.

## Methods

### Participants and procedures

The study involved a real-world, micro-longitudinal design across 42 days, assessing a cohort of full-time financial traders (*n* = 17) employed by a proprietary trading company in New York City. This group, a community sample, was chosen, in part, due to their known real-world utilization of alcohol [33–35] as well as caffeine [24, 25], in isolation and in combination. All individuals met the inclusion criteria, which were: 18 years old or older, fluent in English, and able to complete online surveys daily. Participants could exclude/eliminate themselves from the study at any time by contacting the study administrator. Participants were male (with female participants not being available candidates within the trading organization for which access was granted and available), with the age range of 23 to 44 years old. Their mean age was 30.8 (2.3 [standard deviation]). Eleven subjects (64.7%) self-identified as white, 4 Asian (23.5%), 1 (5.9%) Black and 1 Native American (5.9%). One participant reported taking sleep medication. All reported results remained significant (*p* < 0.05) (or non-significant) whether or not this individual was included in the analyses. All subjects provided written informed consent, with the study approved by UC Berkeley Committee for Protection of Human Subjects guidelines.

At 8:00 a.m. each day during the 6-week survey period, each participant received an automated email inviting them to complete the daily survey using a personalized link. Before the survey began, participants were given explicit instructions asking them to complete the survey the day following the night of sleep as early as possible. In the rare event they were unable to do so (e.g., unable to access the internet), they were asked to complete the survey as soon as possible (within 72 hours) using specific survey links corresponding to each night of sleep. This approach therefore minimized the risk that participants provide identical responses for multiple surveys at once. Participants who missed surveys for more than 2 days in a row received a reminder email from the research team. The surveys were conducted online using the Qualtrics platform. As compensation, subjects who completed at least 95% of daily surveys were entered into a drawing for one of two Amazon.com gift cards of $50 dollars.

### Measures

Participants first completed a baseline survey that included questions about their demographics (e.g., age, gender, race, marital status), general subjective sleep quality on a 100-point scale, and sleep medication status (yes/no). Only recent use of sleep-related medication was examined among the participants. Participants’ use of wake-promoting or stimulant medications that may affect sleep was not specifically assessed. After completing the baseline survey, participants responded to a copy of the daily survey that would be administered across the subsequent 6-week period in order to familiarize themselves with the Qualtrics platform prior to their first longitudinal data entry. Responses to this copy of the daily survey were not included in the data analysis.

The longitudinal daily survey asked subjects to quantify features of their previous night’s sleep, targeting the following measures: subjective sleep quality (assessed using the question, “How well did you sleep last night?” with no other prompts, rated on a 100-point using a visual analogue style slider scale tool), number of night-time awakenings, and sleep duration rounded to the nearest half-hour. In addition, subjects were asked to report the number of cups of caffeinated beverages, and the number of glasses of alcoholic beverages, that they consumed in the previous day/evening, rounded to the nearest 0.5 (e.g., 1.6 cups → 1.5 cups of caffeinated beverages, and the same for glasses of alcohol). Participants were asked to report 8 US ounces of caffeinated beverage as one cup, and one standard drink as one glass of alcoholic beverage. They were briefed on the definition of a standard drink using examples of different alcoholic beverages.

### Data analysis

Basic descriptive statistics were first tabulated for the data collected during the daily survey period (i.e., consumption of alcoholic/caffeinated beverages and the sleep variables). Mean values across the study period for measures of interest (e.g., sleep duration) were first calculated for each subject. Then, a mean of all of the subject-specific mean values was obtained, in order to account for any potential differing response rates between subjects. One subject who submitted only weekday and no weekend responses, and another subject who submitted weekday responses but only one-weekend response, were excluded from analyses involving weekends.

The three predictions emerging from the hypothesis were tested using mixed effects models. Three separate mixed-effects models were created, one for each of the sleep measure dependent variables (subjective sleep quality, sleep duration, and night-time awakenings). The independent variables of interest in the three models were caffeine consumption, alcohol consumption, and the interaction between caffeine and alcohol consumption, corresponding to the three predictions of the experimental model.

Anticipating a substantive difference between average weekday and weekend intake quantities for both alcoholic and caffeinated beverages [29, 30, 36, 37] a weekend dummy variable, alcohol-weekend interaction, and caffeine-weekend interaction were included as control variables. In addition, a one-day lagged dependent variable term (“*lag(DV*, *1)*”) was also added as a control variable, meaning the previous day’s sleep measure was fitted as a predictor of each day’s sleep measure values. The purpose of adding a lagged term was to control for autocorrelation, or the similarity between daily sleep measures as a function of one-day time lag, in the data analysis. Finally, a random intercept (“*(1 | Subject)*”) was added, which assigns a different mean value for each subject in order to account for individual differences in sleep measures. For example, for subjective sleep quality, the mixed effects model was fit as follows:

SubjectiveSleepQuality∼Alcohol+Caffeine+Alcohol:Caffeine+Weekend+Alcohol:Weekend+Caffeine:Weekend+lag(SubjectiveSleepQuality,1)+(1|Subject)

Alpha level of 0.05 was used to assess the statistical significance of the *a priori* dependent variables. Data analysis was performed using RStudio [38] / R [39]. Mixed effects models were fitted using the lme4 R package [40]. Finally, we assessed whether sleep affects the next day’s alcohol and caffeine consumption, given evidence of bidirectional links between substance use and sleep problems [31, 32]. Analyses of the association between the three sleep variables (independent variables) and the following day’s alcohol, and separately, caffeine use quantities (dependable variable) were then assessed using mixed effects models. Weekend dummy variable and the previous day’s alcohol/caffeine use quantity were included in the analyses as covariates.

## Results

### Characteristics of consumption and sleep

In short, participants reported consuming a mean average of 1.14 (0.77) cups of caffeinated beverage and 0.78 (0.85) glasses of alcoholic beverage per day. Average sleep duration was 7.36 (0.53) hours per night, while subjective sleep quality averaged 72.2 (15.1) points on a 100-point scale and the number of night-time awakenings averaged 0.91 (0.6) per night. Additional characteristics and statistics can be found in S1 Appendix (“Descriptive Statistics”).

### The effects of caffeine consumption on sleep

**Fig 1:**
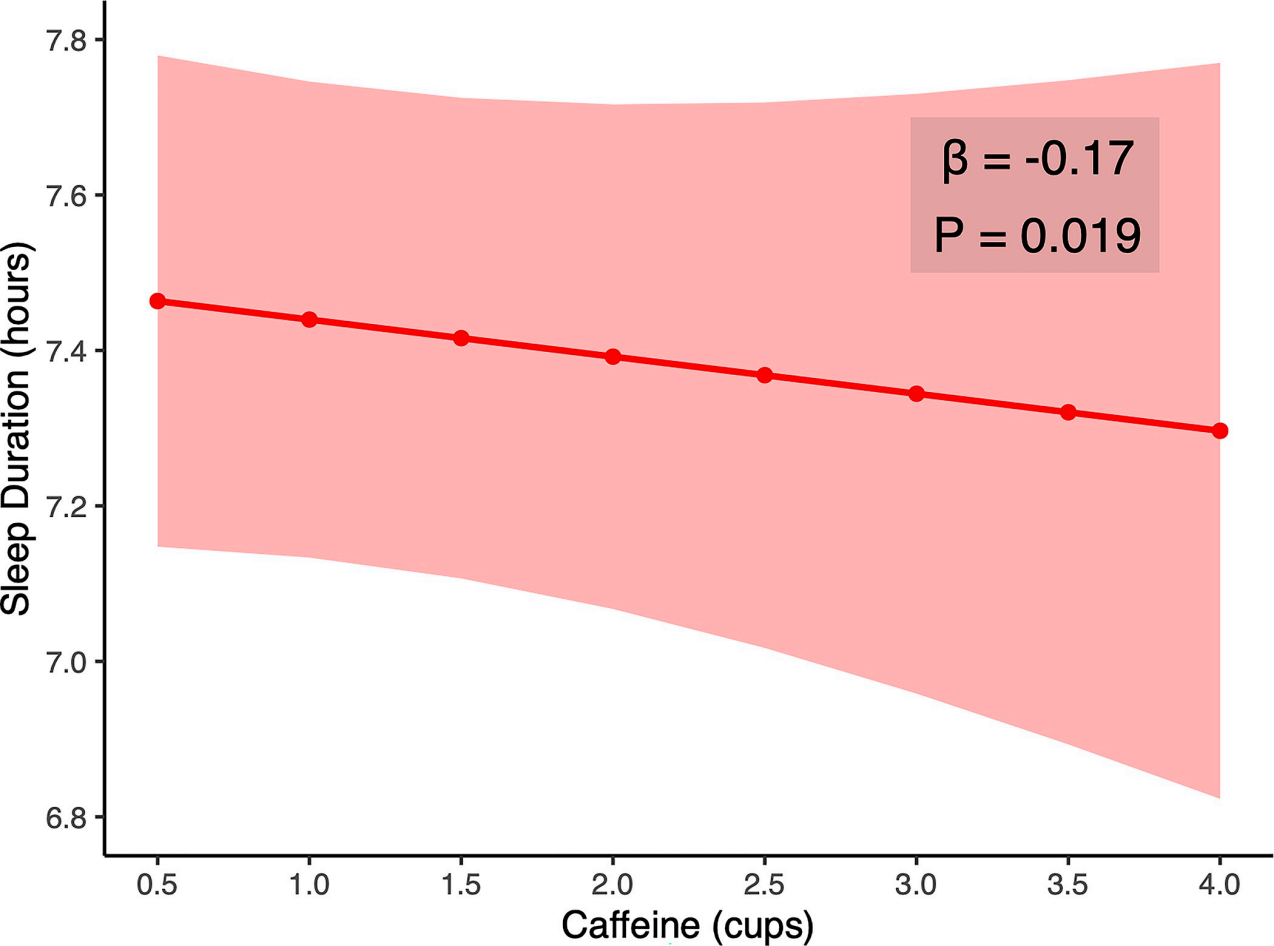
Effect of caffeine on sleep duration. Caffeine consumption was linearly associated with shorter sleep duration in the mixed effects model.

First, examining the hypothesized relationship between daily caffeine intake and sleep quality, a mixed effects model was fit with caffeine consumption, alcohol consumption, and their interactions as independent variables, and subjective sleep quality as the dependent variable. Focusing on sleep quantity, again using a mixed effects model, caffeine consumption was also associated with a substantial decline in sleep quantity, such that the greater the caffeine consumption, the greater the impairment in sleep amount (*t* = -2.35, *p =* 0.019, **Fig 1**), supporting the experimental prediction. Indeed, for every cup of caffeinated beverage consumed, sleep amount decreased by 10.4 minutes. Given that the subjects consumed 1.14 cups of caffeinated beverages per day on average, the model suggests 11.8 minutes of sleep was lost from caffeinated beverage intake each night, translating to over an hour of accumulating sleep debt each week.

Moving beyond sleep quality, based on the expected temporal order, we further examined whether caffeine consumption during the day/evening consequently altered sleep quality. Consistent with prior literature reporting cross-sectional associations, and supporting the experimental hypothesis, caffeine consumption exhibited a directionally consistent but non-significant relationship with lower subjective sleep quality (*t* = -1.60, *p =* 0.11). Caffeine consumption was also not associated with the number of self-reported awakenings (t = -0.54, p = 0.590). Therefore, in this real-world setting among financial traders, caffeine use was most associated with a significant reduction in the ability to obtain a sufficient *quantity* of sleep relative to the aforementioned impact on the *quality* of sleep, implying that caffeine consumption may lead to a sleep-state mismatch in perception between sleep quantity and quality.

### The effects of alcohol on sleep

**Fig 2:**
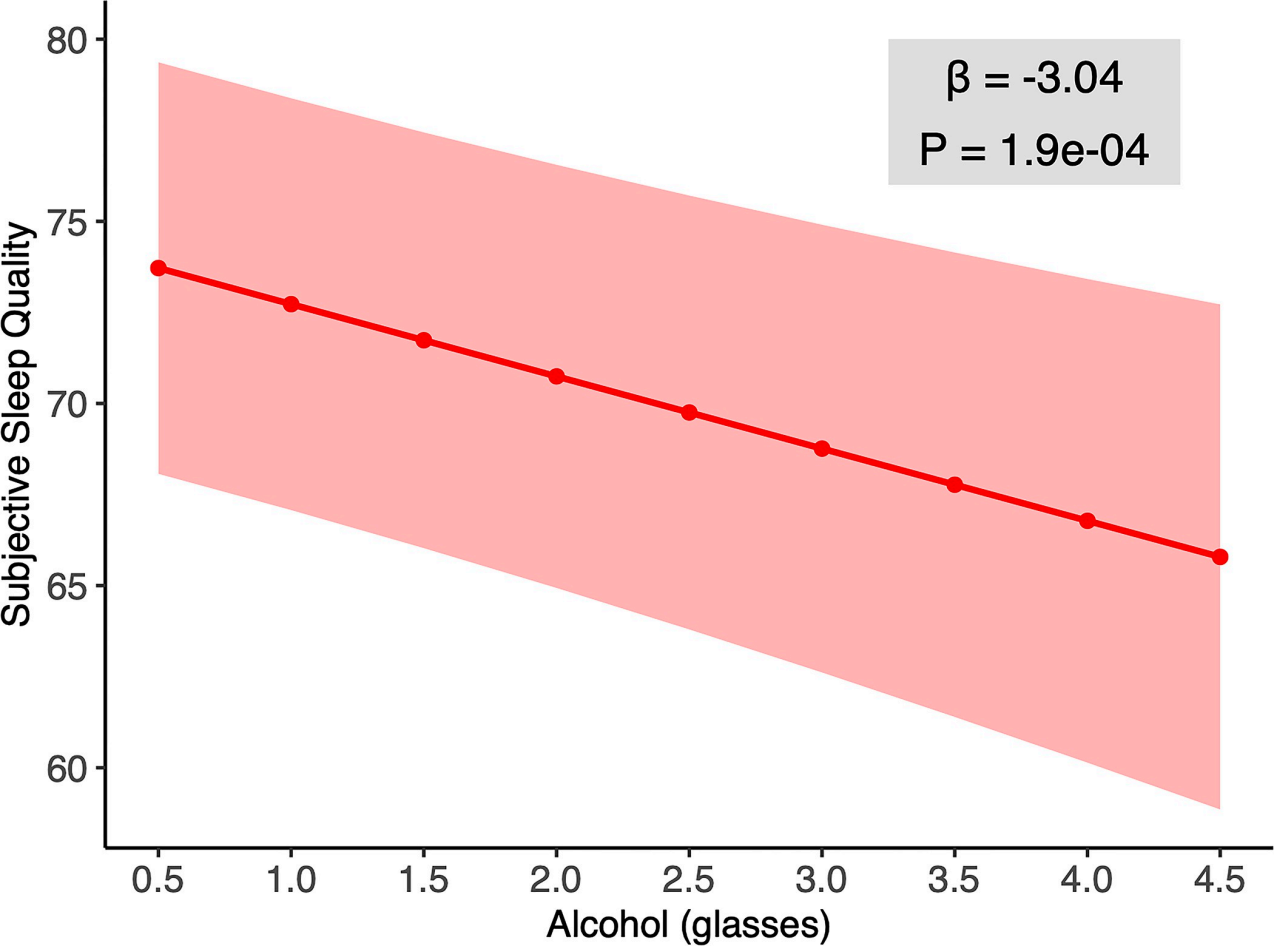
Effect of alcohol on subjective sleep quality. Alcohol consumption was negatively correlated with subjective sleep quality in the mixed effects model.

Having examined the independent influence of caffeine consumption on sleep, we next examined the independent influence of alcohol intake on subsequent night-time sleep. Corroborating the hypothesis prediction, alcohol consumption was associated with lower subjective sleep quality (parameter estimate *t* = -3.76, *p* < 0.001). Each glass of alcohol consumed predicted a decline in subjective sleep quality of 3 points on a 100-point scale the following day. Considering the subjects reported drinking 0.78 glasses of alcohol per night, alcohol consumption was responsible for subjective sleep quality deterioration of, on average, more than 2 points on a 100-point scale (**Fig 2** slope and 95% confidence interval).

With respect to sleep duration, alcohol consumption was not associated with a change in sleep duration (*t* = 0.33, *p =* 0.742), unlike its role in predicting a decline in subjective sleep quality. This finding suggests alcohol’s phenotype of sleep impairment in this cohort, on a night-to-night basis, is unlike that of caffeine, which was linked with a marked decrease in sleep quantity. The final examination targeted the a priori measure of sleep fragmentation, quantified by the number of self-reported night-time awakenings. The association between alcohol consumption and night-time awakenings, while positive (the predicted direction), was statistically insignificant (*t* = 1.71, *p =* 0.089).

### The effects of alcohol-caffeine interaction

**Fig 3:**
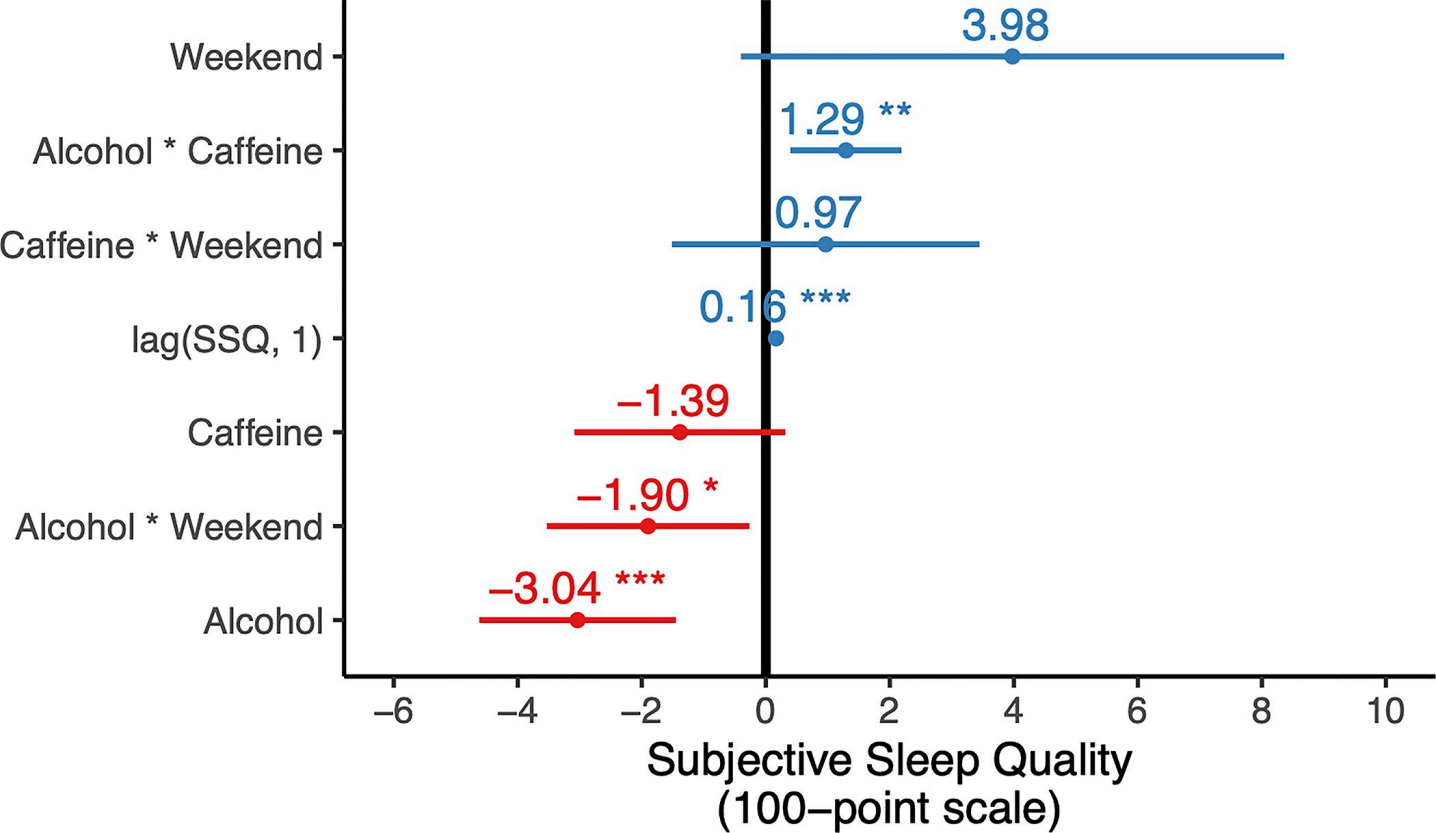
Effects of independent variables on subjective sleep quality. Alcohol consumption was linked to a reduction in subjective sleep quality by over 3 points per glass (on a 100-point scale), while the alcohol-caffeine interaction predicted a small improvement.

Having tested the independent contributions of caffeine and alcohol consumption on sleep, we next sought to examine the *dual*, interacting effects of combined-use caffeine and alcohol on night-time sleep, rather than either alone. Somewhat contrary to the hypothesis prediction, when alcohol was consumed in combination with prior caffeine consumption, the interaction between the two substances had a *positive* effect on subjective sleep *quality* (*t* = 2.83, *p =* 0.005, **Fig 3**). This would suggest that the known sedative influence of alcohol [41–44] may mask the otherwise detrimental psychoactive alerting impact of prior caffeine consumption on overall subjective sleep quality [14–17]. Therefore, the daytime stimulant effects of caffeine and the night-time sedating effects of alcohol may act to leave the subjective perception of sleep quality indifferent. While no past study evaluating the effects of consuming caffeine and alcohol in this temporal order on sleep was identified in our literature search, these results are consistent with past laboratory findings of improved subjective sleep quality following the combined consumption of alcohol and caffeine, relative to alcohol alone [8]. Post-hoc tests from the same study indicated that this increase in subjective quality was the result of improved alertness upon awakening, and not superior quality of sleep the night prior [8].

We next sought to determine the extent of the explanatory power of alcohol and caffeine on subjective sleep quality, before evaluating sleep quantity. Without consideration of either alcohol or caffeine, the mixed effects model containing the weekend dummy variable lagged term (previous night’s subjective sleep quality), and random effect component explained only 3% of night-to-night variations in subjective sleep quality. However, adding both alcohol and caffeine and their interaction terms as predictors tripled the explanatory power of the model from 3% to 10.4%, validating the interaction impact of alcohol and caffeine intake on nightly sleep quality (caffeine alone offered an explanatory power of 3.2%, and alcohol 8.9%).

**Fig 4:**
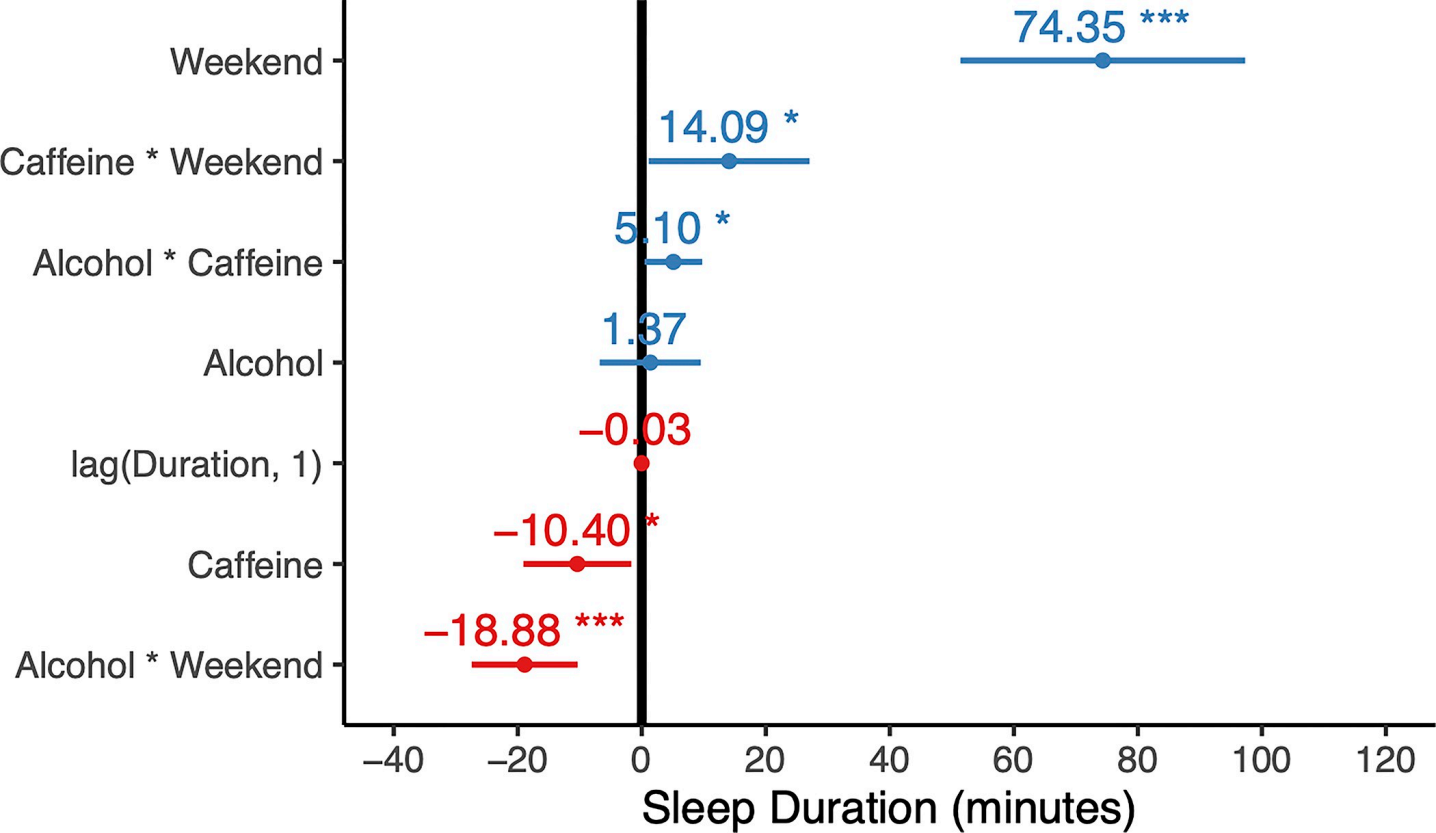
Effects of independent variables on sleep duration. Caffeine consumption predicted a decrease in sleep duration by over ten minutes per cup, while caffeine-alcohol interaction was associated with a small increase.

We next focused on sleep quantity i.e., sleep duration. Analyses demonstrated an interrelationship between caffeine and alcohol that, at first, appears contradictory to the sleep-disrupting effects of each. Specifically, when individuals had consumed caffeine but subsequently consumed alcohol in the evening, the otherwise detrimental impact of caffeine on sleep amount was prevented, leading to a modest increase in overall sleep duration (*t* = 2.16, *p =* 0.032, **Fig 4**) compared to consuming caffeine alone. That is, subsequent consumption of the sedative alcohol following the daytime consumption of the stimulant caffeine placated the otherwise negative impact of caffeine on sleep amount, a temporal interaction effect that has not been identified in past literature. These two factors and their interaction also demonstrated substantial contributions to the mixed effects model’s explanatory power, from 12.5% to 16.7%. That is, knowing the day-to-day caffeine and alcohol consumption history, along with the day of the week and the previous night’s sleep duration explained a sixth of the night-to-night variation in sleep duration in these financial traders.

While the interaction of caffeine and alcohol on sleep duration offered explanatory insights, their interaction also accounted for a modest amount of night-time awakenings. Specifically, including alcohol, caffeine, and their interaction in a similar mixed effects model to those above but focusing on the outcome measure of awakenings at night explained 6.7% of the variance. This value was almost double that of the base model without alcohol, caffeine or their interaction included.

### Assessing bidirectionality: Sleep measures and the following day’s alcohol and caffeine intake

The above analyses characterized the directional association between the consumption of caffeinated and alcoholic beverages during the day and their impact on participants’ subsequent night’s sleep (i.e., day → sleep). A final analysis sought to determine whether a participant’s night of sleep prior (specifically the three a priori variables of sleep quantity, sleep quality, and the number of awakenings) exerted a subsequent associational influence on alcohol and caffeine consumption behaviors in the following day (i.e., sleep → day). For caffeine and for alcohol separately, a mixed effects model was fit with the three sleep measures (subjective sleep quality, sleep duration, and night-time awakenings) as independent variables and the next day’s consumption quantity as the dependent variable.

For caffeine consumption, none of the three sleep variables on the night prior predicted the extent of caffeine intake the next day (all *t* < 0.25, *p* > 0.79). The number of night-time awakenings was not significantly associated with the extent of alcohol consumption the following day, although this effect was directionally consistent with our prediction (*t* = -1.786, *p* = 0.075). Sleep quantity and sleep quality the night prior were not associated with alcohol consumption the following day (both *t* < -0.06, *p* > 0.95).

## Discussion

Using a micro-longitudinal study across several weeks under a free-living condition in a group of financial traders, here, we demonstrate three key findings: 1) within individuals over time, from one night to the next, increases in daily caffeine consumption are significantly associated with reductions in subsequent nightly sleep *quantity*, yet this impairment in the night-time quantity of an individual’s sleep did not translate into perceiving subjective reductions in sleep *quality*, suggesting that caffeine may produce a sleep-state mismatch in perception between sleep quantity and quality, 2) increasing evening-to-evening consumption of alcohol negatively impacts subsequent and consequential night-to-night subjective sleep *quality* (with minimal effects on sleep amount). Furthermore, this same deleterious relationship was observed for sleep fragmentation (night-time awakenings), and 3) the night-time consumption of alcohol minimizes the otherwise detrimental impact of daytime caffeine on sleep, suggesting a potential explanatory path by which the sedative influence of alcohol can modulate the stimulant effects of caffeine, and may explain why individuals habitually self-medicate with night-time alcohol to override the effects of their daytime caffeine intake amount and vice versa [41, 44–48]. We next discuss each of these findings in detail.

### Caffeine consumption reduces sleep quantity but not subjective sleep quality

Daily increases in caffeine consumption significantly accounted for nightly reductions in sleep quantity, consistent with past large-scale cross-sectional studies [49–51], and longitudinal experimental studies in which caffeine intake was manipulated [52–54]. The observed caffeine-induced reduction in sleep duration supports the known function of caffeine as a central nervous system stimulant. The alerting effects of caffeine are, in part, attributed to its role in antagonizing adenosine receptors, which play a key role in regulating sleep homeostasis [55]. During wakeful periods, extracellular adenosine levels gradually rise in response to prolonged neuronal activity, which leads to a reduction in neuronal activity and induces sleep [56]. Caffeine acts to antagonize this process, which also increases the cerebral utilization of noradrenaline, dopamine, and serotonin–all of which play a role in augmenting wakefulness, thereby instigating stimulant-driven alertness [57]. Both offer plausible explanatory mechanisms accounting for why day-to-day changes in caffeine intake, by way of alternating day-to-day adenosine signaling within the brain, resulting in night-to-night impairments in the amount of sleep obtained by the brain.

Our current findings build on past experimental laboratory studies by providing a real-world observational assessment of caffeine on sleep duration in a micro-longitudinal study across multiple days and nights. Such a design enables additional insights into intra-individual variations, beyond cross-sectional inter-individual understanding. Moreover, the current study afforded night-to-night evaluation of the effects of caffeine when accounting for (and examining the interaction between) three additional ecological key factors: 1) person-specific variations, 2) weekday/weekend consumption difference, and 3) alcohol consumption.

In contrast to sleep quantity, there was no such association between caffeine consumption and night-to-night changes in subjective sleep quality in this cohort and study, suggesting a sleep-state misperception. While this may seem antithetical to the known stimulant properties of caffeine, prior reports have similarly described mixed associations between caffeine and subjective sleep quality. While several studies have reported the expected reduction in sleep quality [54, 58, 59], other cross-sectional and longitudinal investigations have found no impact on reported sleep quality [60–62]. One theory explaining the lack of an effect observed in these past reports and in our study is that individuals learn to regulate their caffeine consumption to fit their individual patterns of caffeine response over time. As such, individuals drink an amount that they subjectively recognize is not altering their perceived sleep quality. This in turn is hypothesized to mitigate the negative effects on self-reported sleep quality [45], and may offer explanatory insights into the findings of the current study. Given the demonstrated reduction in sleep quantity from caffeine consumption in our cohort, however, a potential danger from this sleep-state misperception arises. While their sleep quantity was objectively reduced by caffeine, the subjects did not perceive their sleep quality as suffering, suggesting they may not recognize the harm of caffeine on sleep. This mismatch in perception between sleep quantity and quality may contribute to the continued use of caffeine despite its negative consequences on sleep.

### Alcohol consumption negatively affects subjective sleep quality and moderately increases night-time awakenings

Independent of caffeine, subjective sleep quality is consistently rated as lower following alcohol consumption [7, 44, 63, 64]. Our identification of an association between alcohol consumption and impaired sleep quality is consistent with these reports, as is our finding that evening alcohol consumption increases consequential night-time awakenings and thus sleep fragmentation [6, 7, 65]. The current study importantly extends these prior studies by demonstrating that the disruptive effects of alcohol on sleep remain significant on a day-to-day basis in real-world settings. This was true even after controlling for person-specific variations in consumption, previous night’s sleep, consumption difference between weekdays and weekends, and caffeine consumption. Since the choice of drinking alcoholic beverages is a voluntary behavior, our real-world data serve as reaffirming motivational evidence that minimizing alcohol consumption in service of improving sleep is an actionable insight for behaviorally-oriented sleep interventions, and suggests that such interventions would carry a public health benefit.

Mechanistically, the reduction in day-to-day reported sleep quality on nights when alcohol was consumed by these financial traders is likely explained by at least three known physiological effects of ethanol. First, alcohol consumption induces earlier-than-normal slow-wave sleep (SWS) during the first half of sleep [20, 66]. This alcohol-induced alteration in the sleep cycle then negatively impacts sleep quality and increases sleep fragmentation in the second half of sleep when blood alcohol level decreases, potentially due to a reduction in SWS pressure in the latter part of the night [48, 67].

Second, alcohol suppresses REM sleep in the early and middle portions of the sleep period. This can result in homeostatic compensatory efforts by the brain that lead to increases in REM sleep intensity in the later part of the night as blood alcohol level decreases [63, 67]. Such a REM sleep rebound effect is known to cause more frequent night-time awakenings, which, in turn, leads to inferior subjective sleep quality [64, 66, 68, 69].

Finally, alcohol consumption significantly increases activation of the sympathetic nervous system during sleep, while inhibiting the parasympathetic nervous system [70–72]. These changes are linked to higher heart rate, higher blood pressure, and lower heart rate variability during sleep. Additionally, such excessive sympathovagal balance is associated with a greater number of night-time awakenings, the result of which is a reduction in subjective sleep quality [72–75]. Either one of these mechanisms, or a combination of some or all three, offers plausible insights into the observed night-to-night impact of alcohol consumption in this group.

### Caffeine and alcohol interaction found to offset negative effects on sleep

An intriguing interaction was discovered between caffeine and alcohol consumption, such that alcohol consumption at night partially offset caffeine’s otherwise negative impact on sleep duration. However, this is not to imply that alcohol consumption is a useful tool for overcoming the negative effects of excessive caffeine consumption. Rather, the finding suggests that the GABA-receptor-mediated sedating effects of alcohol [41–44] may partially mitigate the stimulant effects of caffeine [14, 45–47] when the two substances are consumed on the same day.

Conversely, we further identified a lessening of the negative impact of alcohol on the next-day subjective evaluation of sleep quality by caffeine. However, this is unlikely due to any beneficial sleep-quality promoting effects of caffeine, but rather, the overcoming of the lower alertness and fatigue caused by alcohol-induced sleep disruption, mediated through caffeine’s alerting effect. Indeed, prior laboratory and cross-sectional survey studies corroborate the role of caffeine in reducing alcohol-induced mental fatigue [21, 76, 77] and decrements in alertness [78, 79]. Therefore, the habitual use of caffeine to “self-medicate” and thus overcome the still-non-restorative alcohol-induced sleep in the morning through greater alertness would appear to be a cogent explanation for this deleterious interaction observed in the current micro-longitudinal investigation. Similarly, routine night-time consumption of alcohol may also be explained as a form of “self-medication” in an effort to alleviate the stimulant effects of daytime caffeine intake based on the interaction results we have identified.

Reversing the directional lens, our final analysis evaluated the link between sleep variables the night prior and caffeine and alcohol consumption behaviors the following day. Previous cross-sectional surveys and experimental studies have indicated a bidirectional relationship between sleep and alcohol use: not only does alcohol predict poorer subsequent sleep, but poor sleep can predict increased subsequent alcohol use [66, 80–82]. For example, across both longitudinal survey findings in adolescents [83] and cross-sectional evaluations of university students [84, 85], poor subjective sleep quality predicted increased use of alcohol.

In contrast to these data, we did not identify a significant relationship between prior sleep duration or subjective sleep quality and next-day’s alcohol consumption in a cohort of working professionals, although the number of prior night-time awakenings did exhibit a near significant association (*p* = 0.07) with next day alcohol use, as would be expected. At least two factors may explain these differences. First, the strength of the significance may have been reduced by merging weekdays with weekends. Indeed, the variable of weekend was a statistically significant predictor of the next day’s alcohol use (*t* = 1.986, *p* = 0.048). Moreover, both average alcohol consumption and sleep duration were significantly higher during weekends than on weekdays (all *p* < 0.001) consistent with past findings [27, 29, 86].

Second, participants in the study may self-regulate their day-to-day alcohol consumption in order to meet their work demands. This may be especially relevant considering that the participants’ profession–stock market trading–demands high levels of attention and cognitive processing speed during fixed hours on weekdays. Thus, individuals may moderate their alcohol intake before weekdays to maintain higher job performance, even when their previous night’s sleep was poor.

Past research has identified a so-called “coffee cycle”, in which feeling tired in the morning caused by prior poor sleep the night before leads to increased daytime caffeine use. This in turn impairs subsequent sleep the following night, and the cycle escalates thereafter in a self-fueling, bidirectional relationship [45, 46, 66]. While we did not identify a significant such relationship cycle wherein nightly sleep predicts next-day caffeine intake, this lack of finding may be explained by the very stable and thus low day-to-day variance in caffeine consumption, which negates the opportunity for an identified relationship.

### Study limitations

Our findings must be appreciated in the context of several limitations. First, although the study was a within-subjects, micro-longitudinal design, our sample was still modest in size and consisted of 100% male-identifying subjects. Second, the sample was biased in the sense that all subjects were working adults in an urban area who work together in the same field (financial trading). While our findings are largely consistent with predictions made by the extant literature, including large epidemiology studies in the general population, the generalizability may nevertheless be limited because of the selective population studied. Third, the study did not evaluate the effects of caffeine and alcohol on sleep in a controlled laboratory setting, but rather, in a real-world ecological setting where uncontrolled factors may have exerted unmeasured effects. Fourth, caffeine quantity was measured in cups of caffeinated beverages instead of caffeine quantity. That said, a random intercept was added to account for individual differences in the relationships between independent variables and the dependent variable, which partially mitigates the issue of caffeine quantity intake standardization at the interindividual level. Fifth, we were unable to assess the impacts of caffeine and alcohol on sleep latency onset in the current study due to limited survey opportunities with the participants. Sixth, the exclusion criteria did not include treatment seekers for sleep-related disorders, alcohol or drug use disorder, or mental health-related disorder, and thus the possibility of interaction effects with these disorders remains unknown. Finally, subjective reports of sleep and awakenings were used rather than objective sleep measurements, such as polysomnography. While past studies have demonstrated high degrees of consistency between subjective sleep measures and polysomnographic data [87–89], the addition of objective sleep measures would have provided stronger support for the dynamics reported, and it is also possible that biases such as recall or social desirability bias could have affected the data reported by the subjects.

### Implications

Taken together, our findings in a cohort of financial traders suggest the sedating effects of alcohol and psychoactive stimulant effects of caffeine obscure each other’s impact on sleep quantity and sleep quality, respectively–potentially explaining their interdependent use (i.e., “self-medication” of evening sedation with alcohol to combat the prior daytime ingestion of caffeine and vice versa). More broadly, these results contribute to a unique understanding of the singular and combinatory impacts of two of the most commonly used substances for augmenting human consciousness under free-living, real-world conditions, the performance-impairing (and thus economic-cost) consequences of which may be important to the business sector and the society.

## Supporting information

S1 Appendix Descriptive statistics. (DOCX)

## References

[1] Global Health Observatory. World Health Organization. http://apps.who.int/gho/data/node.main.602?lang=en. Published 2018.

[2] Chawla J. Neurologic Effects of Caffeine: Overview, Consumption of Caffeine, Physiologic Effects of Caffeine. *Medscape*. 2018. https://emedicine.medscape.com/article/1182710-overview#a2.

[3] Bordeaux B, Lieberman HR. Benefits and risks of caffeine and caffeinated beverages. UpToDate. Published 2022.

[4] Kunst A. Amount of tea drank per day at home in the United Kingdom (UK) in 2019. Statista. https://www.statista.com/statistics/681635/tea-consumption-daily-amount-united-kingdom-uk/. Published 2022.

[5] Rakastettu ja vihattu suomalainen kahvi–Paulig pureutuu kansainvälisenä kahvipäivänä 1.10. kahvikulttuurin myytteihin. Paulig Group. https://www.pauliggroup.com/fi/uutishuone/rakastettu-ja-vihattu-suomalainen-kahvi-paulig-pureutuu-kansainvalisena-kahvipaivana-110. Published 2018.

[6] Arnedt JT, Rohsenow DJ, Almeida AB, et al. Sleep Following Alcohol Intoxication in Healthy, Young Adults: Effects of Sex and Family History of Alcoholism. *Alcoholism*: *Clinical and Experimental Research*. 2011;35(5):870–878. doi: 10.1111/j.1530-0277.2010.01417.x 21323679 PMC3083467

[7] Feige B, Gann H, Brueck R, et al. Effects of alcohol on polysomnographically recorded sleep in healthy subjects. *Alcoholism*, *Clinical and Experimental Research*. 2006;30(9):1527–1537. doi: 10.1111/j.1530-0277.2006.00184.x 16930215

[8] Rohsenow DJ, Howland J, Alvarez L, et al. Effects of caffeinated vs. non-caffeinated alcoholic beverage on next-day hangover incidence and severity, perceived sleep quality, and alertness. *Addictive Behaviors*. 2014;39(1):329–332. doi: 10.1016/j.addbeh.2013.09.008 24090620 PMC3864634

[9] Rouhani S, Tran G, Leplaideur F, Durlach J, Poenaru S. EEG effects of a single low dose of ethanol on afternoon sleep in the nonalcohol-dependent adult. *Alcohol*. 1989;6(1):87–90. doi: 10.1016/0741-8329(89)90078-5 2719820

[10] Chee MWL, Chuah YML. Functional neuroimaging and behavioral correlates of capacity decline in visual short-term memory after sleep deprivation. *Proceedings of the National Academy of Sciences*. 2007;104(22):9487–9492. doi: 10.1073/pnas.0610712104 17517619 PMC1874228

[11] Frenda SJ, Fenn KM. Sleep Less, Think Worse: The Effect of Sleep Deprivation on Working Memory. *Journal of Applied Research in Memory and Cognition*. 2016;5(4):463–469. doi: 10.1016/j.jarmac.2016.10.001

[12] Harrison Y, Horne JA. The impact of sleep deprivation on decision making: A review. *Journal of Experimental Psychology*: *Applied*. 2000;6(3):236–249. doi: 10.1037//1076-898x.6.3.236 11014055

[13] Hudson AN, Van Dongen HPA, Honn KA. Sleep deprivation, vigilant attention, and brain function: a review. *Neuropsychopharmacology*. 2019;45(1):21–30. doi: 10.1038/s41386-019-0432-6 31176308 PMC6879580

[14] Clark I, Landolt HP. Coffee, caffeine, and sleep: A systematic review of epidemiological studies and randomized controlled trials. *Sleep Medicine Reviews*. 2017;31:70–78. doi: 10.1016/j.smrv.2016.01.006 26899133

[15] Hicks RA, Hicks GJ, Reyes JR, Cheers Y. Daily caffeine use and the sleep of college students. *Bulletin of the Psychonomic Society*. 1983;21(1):24–25. doi: 10.3758/bf03329943

[16] Hindmarch I, Rigney U, Stanley N, Quinlan P, Rycroft J, Lane J. A naturalistic investigation of the effects of day-long consumption of tea, coffee and water on alertness, sleep onset and sleep quality. *Psychopharmacology*. 2000;149(3):203–216. doi: 10.1007/s002130000383 10823400

[17] Landolt HP, Werth E, Borbély AA, Dijk DJ. Caffeine intake (200 mg) in the morning affects human sleep and EEG power spectra at night. *Brain Research*. 1995;675(1–2):67–74. doi: 10.1016/0006-8993(95)00040-w 7796154

[18] Mniszek DH. Brighton Sleep Survey: A Study of Sleep in 20–45-Year Olds. *Journal of International Medical Research*. 1988;16(1):61–65. doi: 10.1177/030006058801600107 3350205

[19] Aepli A, Kurth S, Tesler N, Jenni O, Huber R. Caffeine Consuming Children and Adolescents Show Altered Sleep Behavior and Deep Sleep. *Brain Sciences*. 2015;5(4):441–455. doi: 10.3390/brainsci5040441 26501326 PMC4701022

[20] Peacock A, Bruno R, Martin FH. The Subjective Physiological, Psychological, and Behavioral Risk-Taking Consequences of Alcohol and Energy Drink Co-Ingestion. *Alcoholism*: *Clinical and Experimental Research*. 2012;36(11):2008–2015. doi: 10.1111/j.1530-0277.2012.01820.x 22897756

[21] Drake CL, Roehrs T, Turner L, Scofield HM, Roth T. Caffeine Reversal of Ethanol Effects on the Multiple Sleep Latency Test, Memory, and Psychomotor Performance. *Neuropsychopharmacology*. 2002;28(2):371–378. doi: 10.1038/sj.npp.1300026 12589390

[22] Pennay A, Lubman DI. Alcohol and energy drinks: a pilot study exploring patterns of consumption, social contexts, benefits and harms. *BMC Research Notes*. 2012;5(1). doi: 10.1186/1756-0500-5-369 22824297 PMC3478984

[23] Bush DM, Lipari RN. *Substance Use and Substance Use Disorder by Industry*. Substance Abuse and Mental Health Services Administration Reports; 2015. 26913332

[24] CareerBuilder. CareerBuilder and Dunkin’ Donuts Survey Reveals Which Professions Need Coffee the Most. PR Newswire. https://www.prnewswire.com/news-releases/careerbuilder-and-dunkin-donuts-survey-reveals-which-professions-need-coffee-the-most-130690543.html. Published 2011.

[25] Here are the professions that glug the most coffee. Journalists are officially the biggest addicts. Pressat. https://pressat.co.uk/blog/2014/09/here-are-the-professions-that-glug-the-most-coffee-journalists-are-officially-the-biggest-addicts/. Published 2014.

[26] Nofsinger JR, Shank CA. DEEP sleep: The impact of sleep on financial risk taking. *Review of Financial Economics*. 2019;37(1):92–105. doi: 10.1002/rfe.1034

[27] Haines PS, Hama MY, Guilkey DK, Popkin BM. Weekend Eating in the United States Is Linked with Greater Energy, Fat, and Alcohol Intake. *Obesity Research*. 2003;11(8):945–949. doi: 10.1038/oby.2003.130 12917498

[28] Hasler BP, Dahl RE, Holm SM, et al. Weekend–weekday advances in sleep timing are associated with altered reward-related brain function in healthy adolescents. *Biological Psychology*. 2012;91(3):334–341. doi: 10.1016/j.biopsycho.2012.08.008 22960270 PMC3490026

[29] Lau-Barraco C, Braitman AL, Linden-Carmichael AN, Stamates AL. Differences in weekday versus weekend drinking among nonstudent emerging adults. *Experimental and Clinical Psychopharmacology*. 2016;24(2):100–109. doi: 10.1037/pha0000068 26901592 PMC4828908

[30] Putilov AA, Sveshnikov DS, Bakaeva ZB, et al. Differences in Body Esteem and Sexual Assertiveness between Male and Female College Students. *Journal of Adolescence*. 2021;88:84–96. doi: 10.37506/mlu.v21i4.3131 33667792

[31] Conroy DA, Arnedt JT. Sleep and Substance Use Disorders: An Update. *Current Psychiatry Reports*. 2014;16(10). doi: 10.1007/s11920-014-0487-3 25135784

[32] Pieters S, Burk WJ, Van der Vorst H, Dahl RE, Wiers RW, Engels RCME. Prospective Relationships Between Sleep Problems and Substance Use, Internalizing and Externalizing Problems. *Journal of Youth and Adolescence*. 2014;44(2):379–388. doi: 10.1007/s10964-014-0213-9 25385390

[33] Kahn H, Cooper CL. Stress Amongst Financial Dealers in the City of London. *Handbook of Stress in the Occupations*. 2011:339–355. doi: 10.4337/9780857931153.00046

[34] Kahn H, Cooper CL. Mental Health, Job Satisfaction, Alcohol Intake and Occupational Stress among Dealers in Financial Markets. In: Cooper CL, ed. Managerial, Occupational and Organizational Stress Research. Routledge; 2018:211–224. doi: 10.4324/9781315196244

[35] Oberlechner T, Nimgade A. Work stress and performance among financial traders. *Stress and Health*. 2005;21(5):285–293. doi: 10.1002/smi.1063

[36] Driver EM, Gushgari A, Chen J, Halden RU. Alcohol, nicotine, and caffeine consumption on a public U.S. university campus determined by wastewater-based epidemiology. *Science of The Total Environment*. 2020;727:138492. doi: 10.1016/j.scitotenv.2020.138492 32334214 PMC10292129

[37] Tran NL, Barraj LM, Bi X, Jack MM. Trends and patterns of caffeine consumption among US teenagers and young adults, NHANES 2003–2012. *Food and Chemical Toxicology*. 2016;94:227–242. doi: 10.1016/j.fct.2016.06.007 27288929

[38] RStudio Team. RStudio | Open source & professional software for data science teams. rstudio.com. http://www.rstudio.com/. Published 2020.

[39] R Core Team. R: A language and environment for statistical computing. R Foundation for Statistical Computing. https://www.r-project.org/. Published 2022.

[40] Bates D, Mächler M, Bolker B, Walker S. Fitting linear mixed-effects models using lme4. *Journal of Statistical Software*. 2015;67(1). doi: 10.18637/jss.v067.i01

[41] Chung T, Martin CS. Subjective Stimulant and Sedative Effects of Alcohol During Early Drinking Experiences Predict Alcohol Involvement in Treated Adolescents. *Journal of Studies on Alcohol and Drugs*. 2009;70(5):660–667. doi: 10.15288/jsad.2009.70.660 19737489 PMC2741546

[42] Fang T, Dong H, Xu XH, et al. Adenosine A2A receptor mediates hypnotic effects of ethanol in mice. *Scientific Reports*. 2017;7(1). doi: 10.1038/s41598-017-12689-6 28978989 PMC5627250

[43] Valenzuela CF. Alcohol and Neurotransmitter Interactions. *Alcohol Health and Research World*. 1997;21(144). 15704351 PMC6826822

[44] Vitiello MV. Sleep, alcohol and alcohol abuse. *Addiction Biology*. 1997;2(2):151–158. doi: 10.1080/13556219772697 26735632

[45] O’Callaghan F, Muurlink O, Reid N. Effects of caffeine on sleep quality and daytime functioning. *Risk Management and Healthcare Policy*. 2018;Volume 11(1):263–271. doi: 10.2147/RMHP.S156404 30573997 PMC6292246

[46] Snel J, Lorist MM. Effects of caffeine on sleep and cognition. *Progress in Brain Research*. 2011;190:105–117. doi: 10.1016/B978-0-444-53817-8.00006-2 21531247

[47] Yacoubi ME, Ledent C, Ménard JF, Parmentier M, Costentin J, Vaugeois JM. The stimulant effects of caffeine on locomotor behaviour in mice are mediated through its blockade of adenosine A2A receptors. *British Journal of Pharmacology*. 2000;129(7):1465–1473. doi: 10.1038/sj.bjp.0703170 10742303 PMC1571962

[48] Chakravorty S, Chaudhary NS, Brower KJ. Alcohol Dependence and Its Relationship With Insomnia and Other Sleep Disorders. *Alcoholism*: *Clinical and Experimental Research*. 2016;40(11):2271–2282. doi: 10.1111/acer.13217 27706838 PMC7486899

[49] Higbee MR, Gipson CS, El-Saidi M. Caffeine Consumption Habits, Sleep Quality, Sleep Quantity, and Perceived Stress of Undergraduate Nursing Students. *Nurse Educator*. 2022;47(2):120–124. doi: 10.1097/NNE.0000000000001062 34366419

[50] Halldorsson TI, Kristjansson AL, Thorisdottir I, et al. Caffeine exposure from beverages and its association with self-reported sleep duration and quality in a large sample of Icelandic adolescents. *Food and Chemical Toxicology*. 2021;157:112549. doi: 10.1016/j.fct.2021.112549 34509583

[51] Lodato F, Araújo J, Barros H, et al. Caffeine intake reduces sleep duration in adolescents. *Nutrition Research*. 2013;33(9):726–732. doi: 10.1016/j.nutres.2013.06.005 24034572

[52] Höfer I, Bättig K. Cardiovascular, behavioral, and subjective effects of caffeine under field conditions. *Pharmacology Biochemistry and Behavior*. 1994;48(4):899–908. doi: 10.1016/0091-3057(94)90198-8 7972294

[53] James JE. Acute and Chronic Effects of Caffeine on Performance, Mood, Headache, and Sleep. *Neuropsychobiology*. 1998;38(1):32–41. doi: 10.1159/000026514 9701720

[54] Sin CW, Ho JS, Chung JW. Systematic review on the effectiveness of caffeine abstinence on the quality of sleep. *Journal of Clinical Nursing*. 2009;18(1):13–21. doi: 10.1111/j.1365-2702.2008.02375.x 19120728

[55] Ribeiro JA, Sebastião AM. Caffeine and adenosine. *Journal of Alzheimer’s disease*. 2010;20:S3–15. doi: 10.3233/JAD-2010-1379 20164566

[56] Porkka-Heiskanen T, Kalinchuk AV. Adenosine as a sleep factor. *Sleep and Biological Rhythms*. 2011;9:18–23. doi: 10.1111/j.1479-8425.2010.00472.x

[57] Nehlig A, Daval JL, Debry G. Caffeine and the central nervous system: mechanisms of action, biochemical, metabolic and psychostimulant effects. *Brain Research Reviews*. 1992;17(2):139–170. doi: 10.1016/0165-0173(92)90012-b 1356551

[58] Calamaro CJ, Mason TBA, Ratcliffe SJ. Adolescents Living the 24/7 Lifestyle: Effects of Caffeine and Technology on Sleep Duration and Daytime Functioning. *Pediatrics*. 2009;123(6):e1005–e1010. doi: 10.1542/peds.2008-3641 19482732

[59] Orbeta RL, Overpeck MD, Ramcharran D, Kogan MD, Ledsky R. High caffeine intake in adolescents: associations with difficulty sleeping and feeling tired in the morning. *The Journal of Adolescent Health*. 2006;38(4):451–453. doi: 10.1016/j.jadohealth.2005.05.014 16549311

[60] Del Brutto OH, Mera RM, Zambrano M, Castillo PR. Caffeine intake has no effect on sleep quality in community dwellers living in a rural Ecuadorian village (The Atahualpa Project). *Sleep Science*. 2016;9(1):35–39. doi: 10.1016/j.slsci.2015.12.003 27217907 PMC4866974

[61] Ho SC, Chung JWY. The effects of caffeine abstinence on sleep: A pilot study. *Applied Nursing Research*. 2013;26(2):80–84. doi: 10.1016/j.apnr.2012.08.004 23218455

[62] Janson C, Gislason T, De Backer W, et al. Prevalence of Sleep Disturbances Among Young Adults in Three European Countries. *Sleep*. 1995;18(7):589–597. doi: 10.1093/sleep/18.7.589 8552930

[63] Ebrahim IO, Shapiro CM, Williams AJ, Fenwick PB. Alcohol and Sleep I: Effects on Normal Sleep. *Alcoholism*: *Clinical and Experimental Research*. 2013;37(4):539–549. doi: 10.1111/acer.12006 23347102

[64] Thakkar MM, Sharma R, Sahota P. Alcohol disrupts sleep homeostasis. *Alcohol*. 2015;49(4):299–310. doi: 10.1016/j.alcohol.2014.07.019 25499829 PMC4427543

[65] Peters TJ, Millward LM, Foster J. Quality of life in alcohol misuse: comparison of men and women. *Archives of Women’s Mental Health*. 2003;6(4):239–243. doi: 10.1007/s00737-003-0012-x 14628175

[66] Roehrs T, Roth T. Sleep, sleepiness, sleep disorders and alcohol use and abuse. *Sleep Medicine Reviews*. 2001;5(4):287–297. doi: 10.1053/smrv.2001.0162 12530993

[67] Koob GF, Colrain IM. Alcohol use disorder and sleep disturbances: a feed-forward allostatic framework. *Neuropsychopharmacology*. 2019;45(141). doi: 10.1038/s41386-019-0446-0 31234199 PMC6879503

[68] Roehrs T, Yoon J, Roth T. Nocturnal and next-day effects of ethanol and basal level of sleepiness. *Human Psychopharmacology*: *Clinical and Experimental*. 1991;6(4):307–311. doi: 10.1002/hup.470060407

[69] Williams DL, MacLean AW, Cairns J. Dose-response effects of ethanol on the sleep of young women. *Journal of Studies on Alcohol*. 1983;44(3):515–523. doi: 10.15288/jsa.1983.44.515 6645531

[70] de Zambotti M, Forouzanfar M, Javitz H, et al. Impact of evening alcohol consumption on nocturnal autonomic and cardiovascular function in adult men and women: a dose–response laboratory investigation. *Sleep*. 2020;44(1). doi: 10.1093/sleep/zsaa135 32663278 PMC7819834

[71] Greenlund IM, Cunningham HA, Tikkanen AL, et al. Morning sympathetic activity after evening binge alcohol consumption. *American Journal of Physiology-Heart and Circulatory Physiology*. 2021;320(1):H305–H315. doi: 10.1152/ajpheart.00743.2020 33185112 PMC7864252

[72] Sagawa Y, Kondo H, Matsubuchi N, et al. Alcohol Has a Dose-Related Effect on Parasympathetic Nerve Activity During Sleep. *Alcoholism*: *Clinical and Experimental Research*. 2011;35(11):2093–2100. doi: 10.1111/j.1530-0277.2011.01558.x 21848959

[73] Irwin MR, Valladares EM, Motivala S, Thayer JF, Ehlers CL. Association Between Nocturnal Vagal Tone and Sleep Depth, Sleep Quality, and Fatigue in Alcohol Dependence. *Psychosomatic Medicine*. 2006;68(1):159–166. doi: 10.1097/01.psy.0000195743.60952.00 16449427

[74] Pietilä J, Helander E, Korhonen I, Myllymäki T, Kujala UM, Lindholm H. Acute Effect of Alcohol Intake on Cardiovascular Autonomic Regulation During the First Hours of Sleep in a Large Real-World Sample of Finnish Employees: Observational Study. *JMIR Mental Health*. 2018;5(1):e23. doi: 10.2196/mental.9519 29549064 PMC5878366

[75] Seravalle G, Mancia G, Grassi G. Sympathetic Nervous System, Sleep, and Hypertension. *Current Hypertension Reports*. 2018;20(9). doi: 10.1007/s11906-018-0874-y 29980938

[76] Liguori A, Robinson JH. Caffeine antagonism of alcohol-induced driving impairment. *Drug and Alcohol Dependence*. 2001;63(2):123–129. doi: 10.1016/s0376-8716(00)00196-4 11376916

[77] Marczinski CA, Fillmore MT, Henges AL, Ramsey MA, Young CR. Effects of energy drinks mixed with alcohol on information processing, motor coordination and subjective reports of intoxication. *Experimental and Clinical Psychopharmacology*. 2012;20(2):129–138. doi: 10.1037/a0026136 22023670 PMC3288788

[78] McKetin R, Coen A, Kaye S. A comprehensive review of the effects of mixing caffeinated energy drinks with alcohol. *Drug and Alcohol Dependence*. 2015;151:15–30. doi: 10.1016/j.drugalcdep.2015.01.047 25861944

[79] Smith AP. Effects of caffeine and alcohol on mood and performance changes following consumption of lager. *Psychopharmacology*. 2013;227(4):595–604. doi: 10.1007/s00213-013-2991-2 23377024

[80] Hasler BP, Soehner AM, Clark DB. Sleep and circadian contributions to adolescent alcohol use disorder. *Alcohol*. 2015;49(4):377–387. doi: 10.1016/j.alcohol.2014.06.010 25442171 PMC4424185

[81] Hussain J, Ling L, Stranges S, Anderson KK. Sleep difficulties and alcohol use behaviors in adolescents and young adults: a systematic review. *European Journal of Public Health*. 2020;30. doi: 10.1093/eurpub/ckaa166.103531647528

[82] Stein MD, Friedmann PD. Disturbed Sleep and Its Relationship to Alcohol Use. *Substance Abuse*. 2006;26(1):1–13. doi: 10.1300/j465v26n01_01 16492658 PMC2775419

[83] Wong MM, Robertson GC, Dyson RB. Prospective Relationship Between Poor Sleep and Substance-Related Problems in a National Sample of Adolescents. *Alcoholism*: *Clinical and Experimental Research*. 2015;39(2):355–362. doi: 10.1111/acer.12618 25598438 PMC4331208

[84] Digdon N, Landry K. University students’ motives for drinking alcohol are related to evening preference, poor sleep, and ways of coping with stress. *Biological Rhythm Research*. 2013;44(1):1–11. doi: 10.1080/09291016.2011.632235

[85] Kenney SR, LaBrie JW, Hummer JF, Pham AT. Global sleep quality as a moderator of alcohol consumption and consequences in college students. *Addictive Behaviors*. 2012;37(4):507–512. doi: 10.1016/j.addbeh.2012.01.006 22285119 PMC4329778

[86] Rehm J, Sempos CT, Trevisan M. Average Volume of Alcohol Consumption, Patterns of Drinking and Risk of Coronary Heart Disease—A Review. *European Journal of Cardiovascular Prevention & Rehabilitation*. 2003;10(1):15–20. doi: 10.1177/174182670301000104 12569232

[87] Åkerstedt T, Hume K, Minors D, Waterhouse J. The meaning of good sleep: a longitudinal study of polysomnography and subjective sleep quality. *Journal of Sleep Research*. 1994;3(3):152–158. doi: 10.1111/j.1365-2869.1994.tb00122.x 10607120

[88] Kaplan KA, Hirshman J, Hernandez B, et al. When a gold standard isn’t so golden: Lack of prediction of subjective sleep quality from sleep polysomnography. *Biological Psychology*. 2017;123:37–46. doi: 10.1016/j.biopsycho.2016.11.010 27889439 PMC5292065

[89] Kushida CA, Chang A, Gadkary C, Guilleminault C, Carrillo O, Dement WC. Comparison of actigraphic, polysomnographic, and subjective assessment of sleep parameters in sleep-disordered patients. *Sleep Medicine*. 2001;2(5):389–396. doi: 10.1016/s1389-9457(00)00098-8 14592388

