# Supplementary material for: Sleep, alcohol, and caffeine in financial traders
Source: PLoS One. 2023 Nov 8;18(11):e0291675. doi: 10.1371/journal.pone.0291675 (PMC10631622; doi:10.1371/journal.pone.0291675)

# Descriptive Statistics

*Compliance with daily survey protocol*

Across participants, a total of 553 responses to caffeine, alcohol, and sleep-related daily electronic surveys were obtained over the 6 weeks of the micro-longitudinal study. The mean completion rate was 77.5% (23.6% [standard deviation]). The completion rate was 81.6% (22.5%) for weekdays and 67.2% (32.2%) for weekends. Completion rates were not significantly correlated with the demographic factors we assessed (including age) or with alcohol or caffeine consumption quantities. Regarding daily response timing, out of 553 daily survey responses, 518 (93.7%) were completed during the day following the night of sleep, and 35 (6.3%) were completed afterwards, up to 72 hours later. Furthermore, 376 of 553 daily survey responses (68.0%) were filled out in the morning hours (before noon), with the remaining surveys (32.0%) completed in the afternoon or evening. Upon re-running analyses using only survey responses completed during the day following the night of sleep, and separately, only responses completed in the morning, all results, for all aspects of the hypotheses tested, that were significant (*p* < 0.05), and all that were non-significant, remained so when this revised analysis approach was conducted.

*Basic Characteristics of Caffeine Consumption*

All (100%) subjects reported consuming caffeinated beverages during the study period. Across all participants, caffeine consumption averaged 1.14 (0.77) cups per day. Caffeine consumption was marginally higher during the week, with a mean of 1.18 (0.81) cups per day relative to weekend consumption with a mean of 1.00 (0.74) though this difference did not reach statistical significance (*t*(15) = 1.83, *p* = 0.088). As noted, one participant did not report any data over weekends, limiting weekday vs. weekend differences to 16 of the 17 participants for those specific analyses.

*Basic Characteristics of Alcohol Consumption*

94% of subjects reported consuming alcoholic beverages across the study period. When our analyses were conducted with data from only the participants who reported alcohol consumption, our findings remained statistically significant (*p* < 0.05) (or insignificant). Participants consumed an average of 0.78 (0.85) glasses of alcohol beverages each day. The distribution of alcohol consumption differed across the 7-day week, with a mean of 0.57 (0.71) glasses a day during 5 working weekdays (Monday to Friday), which then increased to a mean of 1.46 (1.38) glasses each day during weekends, *t*(15) = 3.97, *p* = 0.001. **Figure S1** and **Figure S2** describe these alcohol and caffeine consumption characteristics by the individual subject.

*Characteristics of Sleep Quantity and Quality, independent of Caffeine or Alcohol*

Regarding sleep quantity in hours, individuals slept significantly less during weekdays with a mean of 7.11 (0.63) compared to weekends with a mean of 8.16 (0.84) (*t*(15) = 4.99, *p* < 0.001). The combined mean sleep quantity was 7.36 (0.53).

Independent of sleep quantity, mean subjective sleep quality was 72.2 (15.1) on a 100-point scale (with a higher score indicating better sleep quality). Mean subjective sleep quality during weekdays was 72.3 (14.4), which was not significantly different to that reported on the weekend, with a mean of 73.8 (17.4) (*t*(15) = 0.14, *p* = 0.89).

The number of awakenings per night averaged 0.91 (0.60) across participants, and there was no significant difference between the distribution of awakenings during weekdays with a mean of 0.88 (0.60) relative to weekends with a mean of 0.70 (0.89) (*t*(15) = 0.49, *p* = 0.63).

**Table S1** lists descriptive statistics for all sleep variables as well as alcohol and caffeine consumption, across all subjects.

Table S1: Descriptive statistics on alcohol/caffeine consumption and sleep variables

|  | Mean | SD | Mean (Weekday) | SD (Weekday) | Mean (Weekend) | SD (Weekend) | t-value (df=15) | p-value |
| --- | --- | --- | --- | --- | --- | --- | --- | --- |
| Daily alcoholic beverage consumption | 0.78 | 0.85 | 0.57 | 0.71 | 1.46 | 1.38 | 3.97 | 0.001 |
| Daily caffeinated beverage consumption | 1.14 | 0.77 | 1.18 | 0.81 | 1.00 | 0.74 | 1.83 | 0.088 |
| Sleep duration (hours) | 7.36 | 0.53 | 7.11 | 0.63 | 8.16 | 0.84 | 4.99 | <0.001 |
| Subjective sleep quality (100-point scale, higher is better) | 72.2 | 15.1 | 72.3 | 14.4 | 73.8 | 17.4 | 0.14 | 0.89 |
| Awakenings per night | 0.91 | 0.60 | 0.88 | 0.60 | 0.70 | 0.89 | 0.49 | 0.63 |

#

Figure S1: Alcohol consumption by subject


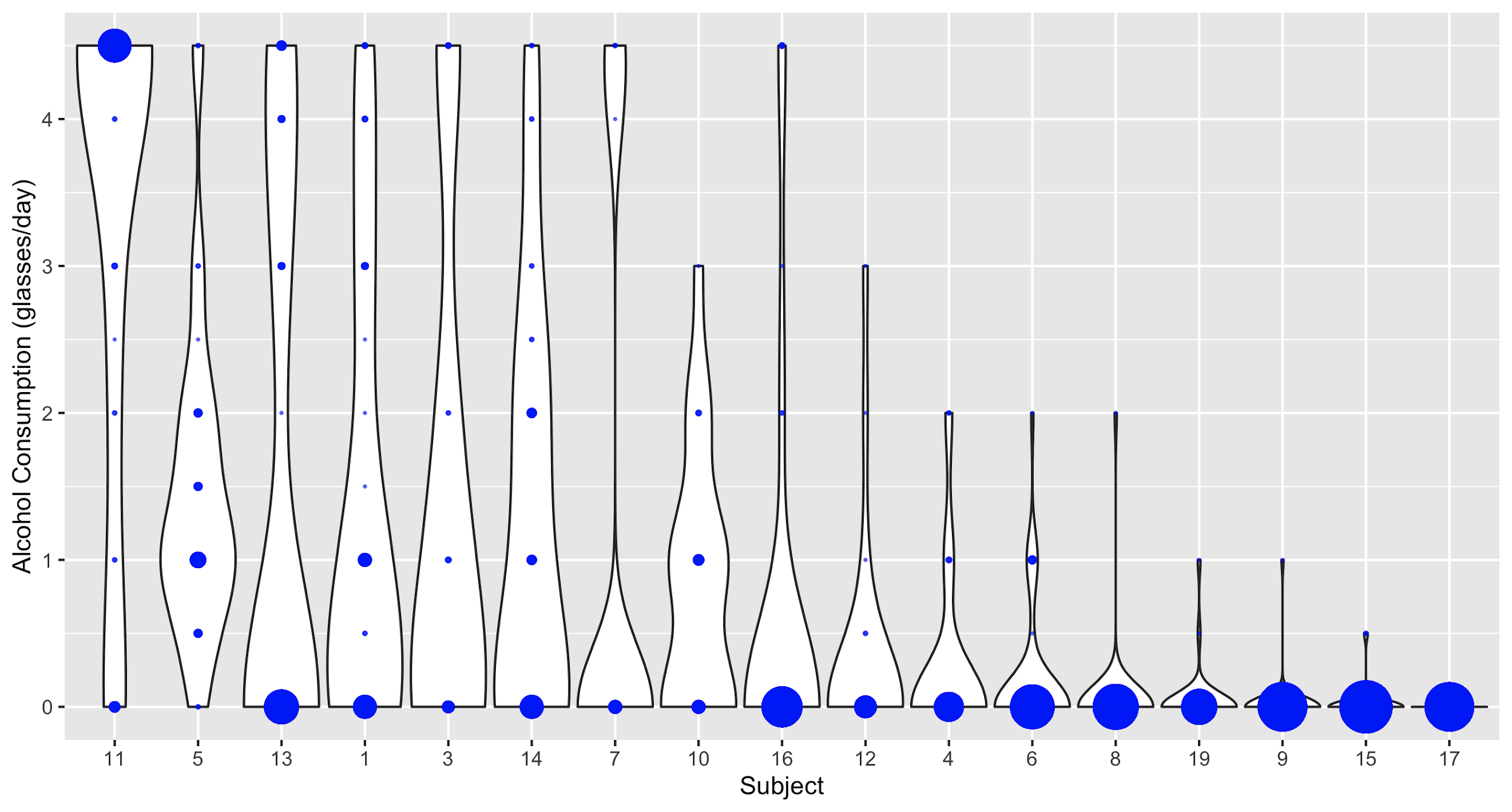


Figure S2: Caffeine consumption by subject


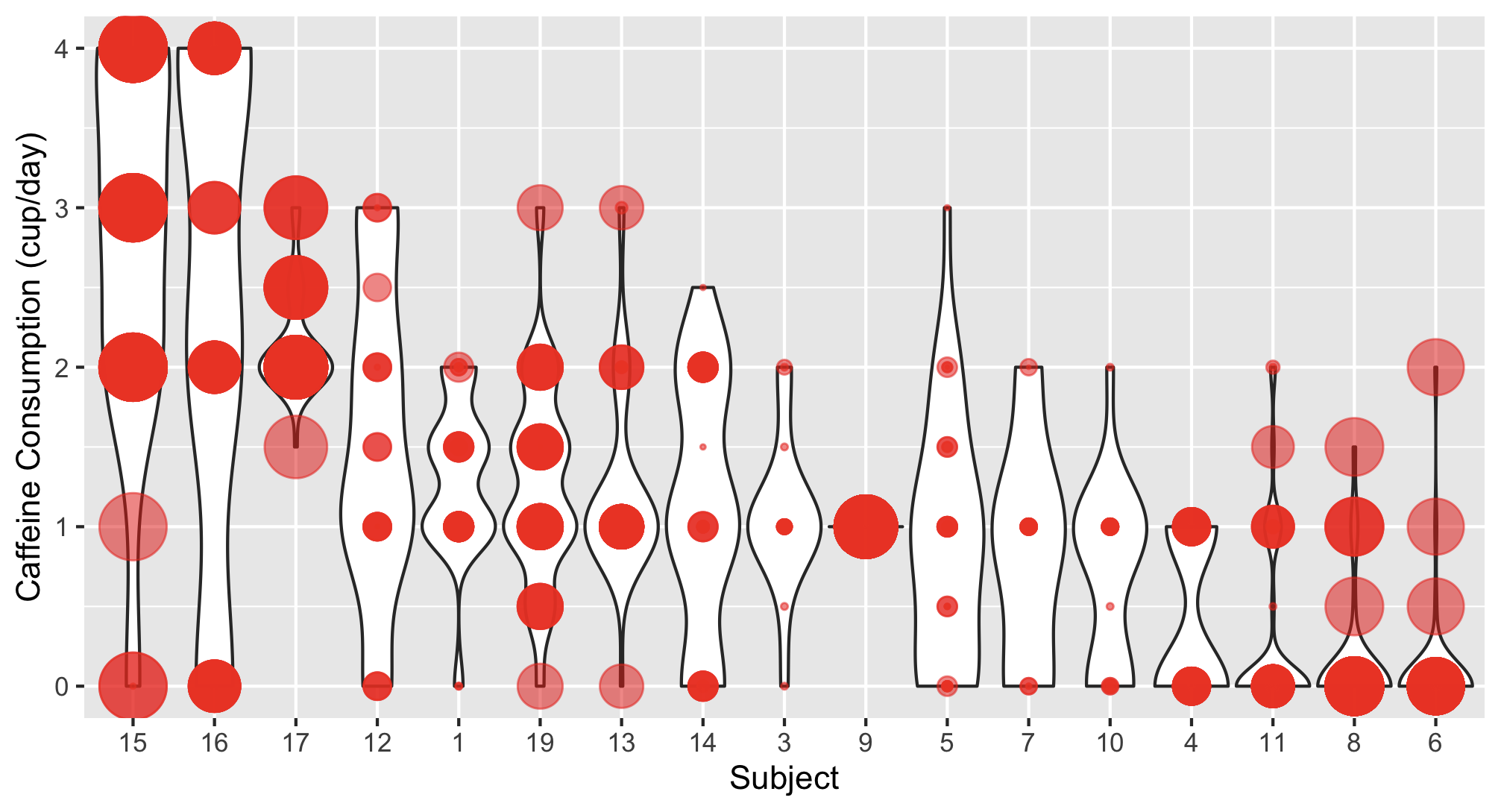

Supplement: S1 Appendix — (DOCX) [file pone.0291675.s001.docx]
